# Cone-Beam Computed Tomography and Histological Findings for Socket Preservation Techniques Using Different Grafting Materials: A Systematic Review

**DOI:** 10.3390/jfb14050282

**Published:** 2023-05-18

**Authors:** Marwa Madi, Ibrahim Almindil, Maria Alrassasi, Doha Alramadan, Osama Zakaria, Adel S Alagl

**Affiliations:** 1 Department of Preventive Dental Sciences, College of Dentistry, Imam Abdulrahman Bin Faisal University, Dammam 32210, Saudi Arabia; aalagl@iau.edu.sa; 2 College of Dentistry, Imam Abdulrahman Bin Faisal University, Dammam 32210, Saudi Arabia; 2160002257@iau.edu.sa (I.A.); 2170005491@iau.edu.sa (M.A.); 2170004599@iau.edu.sa (D.A.); 3 Department of Biomedical Dental Sciences, College of Dentistry, Imam Abdulrahman Bin Faisal University, Dammam 32210, Saudi Arabia; oazakaria@iau.edu.sa

**Keywords:** socket preservation, bone graft, radiology, computer tomography, CBCT, histology, histomorphometric

## Abstract

Objective: Socket preservation techniques have been used to maintain the ridge dimension following tooth extraction. The materials used influence the quality and quantity of newly formed bone. Therefore, the aim of this article was to systematically review the literature reporting both histological and radiographic outcomes of socket preservation techniques after tooth extraction in human subjects. Material and method: A systematic electronic search was performed in the electronic databases. English language clinical studies that were published between 2017 and 2022 and included both histological and radiographic findings for the test and control groups. Our primary search produced 848 articles, and of these, 215 were duplicate studies. A total of 72 articles were then eligible for full-text reading. Results: The review included eight studies that met its inclusion criteria. Three outcomes were compared in the included studies. The percentage of newly formed bone ranged from 21.34 ± 9.14% to more than 50% of new bone formation. The materials that showed more than 50% of newly formed bone formation were demineralized dentin graft, platelet-rich fibrin, freeze-dried bone allograft, corticocancellous porcine, and autogenous bone. Four Studies did not report the percentage of the residual graft materials, while those who reported showed a variable range of a minimum 1.5% to more than 25%. One study did not report the changes in horizontal width at the follow-up period, while other studies ranged from 0.6 mm to 10 mm. Conclusion: Socket preservation represents an efficient technique to preserve the ridge contour with satisfactory newly formed bone in the augmented site and maintaining the vertical and horizontal dimensions of the ridge.

## 1. Introduction

The primary objective of periodontal therapy is to maintain healthy, functional, and aesthetic teeth [1]. Nevertheless, tooth extraction may occasionally be necessary. Implant-supported restorations are one of several prosthetic options available for replacing extracted teeth [2,3].

Since the alveolar process is tooth-dependent, tooth extraction stimulates the bone remodeling process, in which new bone is formed inside the extraction socket while bone resorption occurs on the external socket walls, resulting in significant dimensional changes [4,5].

A study by Paolantonio et al. [6] suggested that placing implants immediately after extraction could minimize the post-extraction ridge changes. However, animal [7,8] and clinical studies [9,10] have shown that immediate implant placement fails to prevent buccal and palatal/lingual bone resorption [11].

To overcome this resorption process, alveolar ridge preservation techniques (ARPs) have been proposed. APR or socket preservation procedure entails the placement of autogenous bone, biological agents, or graft materials into the socket after the extracted tooth has been removed [8,9,10,11,12]. Even though these ARPs limit bone resorption, they do not completely eliminate it [13,14,15]. Socket preservation is a technique-dependent process and may have unpredictable outcomes [8]. The biomaterials used in socket preservation maintain space and promote bone growth primarily because of their osteoconductive properties. Depending on the osteoconductive material used, graft resorption and new bone formation can differ significantly [16,17]. ARP has been studied and recently benefited from the combined use of bromelain [16].

A wide variety of materials have been evaluated, and in many cases, they are more efficient than natural healing only [18,19,20,21,22].

Barone et al. [23] compared natural socket healing versus grafting with corticocancellous porcine bone covered with collagen membrane in a clinical trial. They concluded that using xenograft covered with resorbable collagen membrane decreased the dimensional changes of the extraction socket along with preserving more attached gingiva. 

Chisci et al. [24] compared socket preservation using deproteinized bovine bone versus particulate autogenous bone covered with a collagen membrane. Their results showed no significant difference between the two grafting materials in regard to the horizontal (2.13 ± 0.25, 2.08 ± 0.27 mm, respectively) and vertical bone changes (0.59 ± 0.22, 0.70 ± 0.23 mm, respectively) after 4 months. Vignoletti et al. [25] systematic review observed that the use of grafting material in socket preservation decreased the crestal bone changes following extraction. However, there are no clearly defined guidelines for selecting the type of biomaterial that should be used.

Avila-Ortiz et al. [26] concluded that the most effective materials that decreased horizontal ridge reduction were xenograft and allograft covered by collagen membranes. 

Several grafting materials and biological factors have been used for socket preservation techniques. Despite this, those used materials will influence the quality and quantity of newly formed bone [27]. Thus, the aim of this article was to systematically review the literature reporting both histological and radiographic outcomes of socket preservation after tooth extraction using different grafting materials in human subjects. 

## 2. Materials and Methods

### 2.1. Search Strategy

This study used the following grouping of MeSH terms and keywords in MEDLINE, Scopus, and Web of Science: (“ridge preservation”) OR (“Socket Preservation”) OR “Socket grafting”) OR (“Ridge augmentation”) OR “Socket healing” OR (“Alveolar ridge preservation”).

OR (“ridge change”) AND (“Bone graft”) AND (radiological) OR (“Computer tomography”) OR (radiographically) OR (CBCT) AND (“histology”) OR (“Histomorphometric”). In this study, only human studies published in dental journals were examined. The screening process consisted of three stages conducted by three independent researchers. In case of disagreement regarding the inclusion or exclusion criteria, a discussion was conducted in order to resolve the differences. In the event of a lack of consensus, the decision was made by a third party (a senior researcher).

As part of the first stage, the researchers independently reviewed all titles from the electronic search to determine whether they were relevant. Articles with uncertainty have been included for further evaluation during the next stages. During the second stage, the screening process was conducted independently by the researchers to exclude articles that did not meet the inclusion criteria from the abstracts of the preidentified papers.

The third stage entailed the evaluation of the full-text versions for eligibility. In the third stage, the full text of the articles were evaluated for eligibility.

#### 2.1.1. Protocol

The PRISMA statement (Principles for Reporting Systematic Reviews and Meta-Analyses) [28] was followed for this systematic review. PROSPERO record number 338,380 was assigned to the study protocol. 

#### 2.1.2. Eligibility Criteria

In PICO (population, intervention, comparison, outcomes), we developed the following focus question:

- P: Medically healthy individuals with an indication for teeth extraction.
- I: In socket preservation procedures, the use of graft materials affects both the quantity and quality of newly formed bone.
- C: Different graft materials.
- O: More effective in socket preservation.
- S: Randomized controlled trials (RCTs) and Clinical studies.

#### 2.1.3. Primary Focus Question

Based on histology and cone-beam computed tomography (CBCT) scan results, what are the effects of different graft materials used in socket preservation in medically fit individuals with an indication for teeth extraction?

#### 2.1.4. Main Outcome

(1) The percentage of the newly formed bone. (2) The percentage of the residual graft materials. (3) The changes in horizontal width at the follow-up period

### 2.2. Inclusion Criteria and Exclusion Criteria

The research was included if it involved medically fit adults, was published in English from 2017 to 2022, and evaluated outcome variables using CBCT scans and histological analyses.

Subjects with any contraindication for oral surgery were excluded, as were studies that did not include both a radiological evaluation with CBCT scans and a histological examination. Furthermore, studies that failed to report relevant outcome data or which recorded data in an incompatible format with the outcome variables predetermined in the inclusion criteria were excluded. Furthermore, only third-molar extraction sites, sinus elevation, and immediate implant placement to preserve the alveolar ridge were not included. Studies without follow-up data at or beyond 3 months and those without comparison groups were also excluded.

### 2.3. Data Collection

Study data included both qualitative and quantitative characteristics, including (1) general study characteristics and basic demographic information about subjects (author, year of publication, number of study groups, and number of subjects in each group), (2) performed procedures (flap reflection, material for grafting, application of a barrier membrane, and soft tissue closure), (3) outcome variables of interest (the percentage of the newly formed bone, the percentage of the residual graft materials, and the radiographic dimensional changes at the follow-up period), (4) histological qualitative description, and (5) and histomorphometric findings.

### 2.4. Risk of Bias (Quality) Assessment

Using instructions outlined in the Cochrane handbook for systematic reviews of interventions, the current authors independently assessed each study’s bias risk [29]. Based on the bias evaluation, there were five domains of bias: randomization bias, intervention deviation bias, missing outcome data bias, measurement bias, and selection bias. The study would be classified as “low risk” if all domains are low, “raised some concerns/moderate risk” if at least one domain raised some concerns, and as “high risk” if all domains had high bias risks. The quality risk-of-bias assessment results chart was then generated using the Cochrane Collaboration tool.

## 3. Results

The initial search identified a total of 848 articles from the 3 databases as follows: PubMed: 181 articles; Scopus: 550 articles; and Web of science: 117 articles. A total of 215 were excluded subsequently because of duplicate. A total of 633 were independently screened by reading the title. A total of 248 papers were then excluded after title reading. A total of 385 papers were eligible for abstract independent reading. An amount of 313 articles were then excluded due to being in vitro studies, including sinus elevation procedure, reporting third molar extraction procedure, and of not being a clinical study. The remaining 72 relevant articles were identified for full text reading where the inclusion and exclusion criteria were applied. 

**Figure 1:**
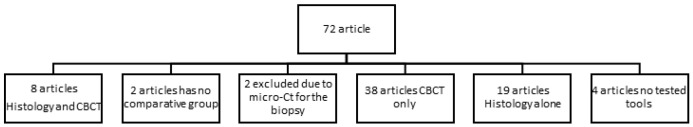
Diagram showing the reason for articles exclusion.

**Figure 2:**
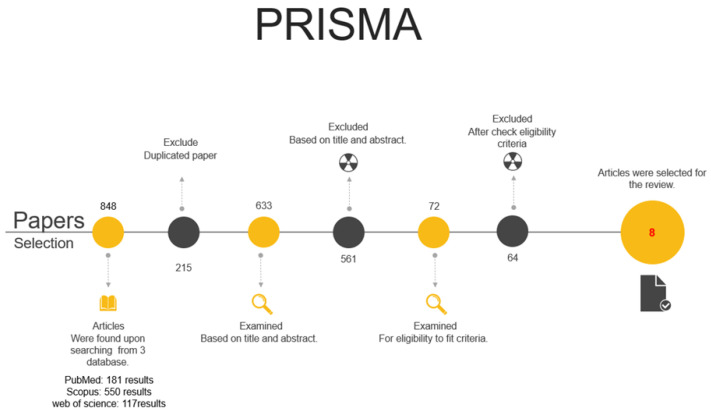
PRISMA displays the search strategy and the screening process leading to the selection of the included eleven trials.

After reading and analyzing the 72 full-text articles, a total of 8 articles [30,31,32,33,34,35,36,37] (Table 1) in which, two of which were not RCTs [33,35], met the inclusion criteria and were selected for this systematic review (Figure 1 and Figure 2).

The κ values for the inter-reviewer agreement of the researchers for potentially relevant articles were >0.9 (titles and abstracts) and >0.9 (full-text articles), indicating an “almost perfect agreement” between the three reviewers. 

### 3.1. Excluded Studies

Following the full-text reading, 63 studies were excluded for the following reasons:

- A total of 57 articles reported either histological or CBCT results.
- One paper evaluated the dimensional changes clinically following the socket preservation procedure [38].
- In one paper, the radiographic findings were reported based on periapical radiographs [39].
- Two papers used the bone graft material to augment the jumping gap after immediate implant placement [40,41].
- No comparative group [42,43], and microcomputed tomography (micro-CT) were conducted only for the bone biopsy [44].

### 3.2. Characteristics of the Outcome Measures

There is a wide range of heterogeneity among the papers included regarding the grafted materials used and the observational period as well.

The materials used included: autogenous whole-tooth graft (AWTG), demineralized dentin graft (ADDG) [30], leukocytes platelet-rich fibrin (L-PRF) or advanced platelet-rich fibrin (A-PRF) [31], moldable poly lactic-co-glycolic acid-coated β-tricalcium phosphate (PLGA-β-TCP), freeze-dried bone allograft (FDBA) [32], demineralized freeze-dried bone allograft (DFDBA) with or without platelet-rich fibrin (PRF) [33], deproteinized bovine bone mineral (DBBM) [38], deproteinized bovine bone with collagen (DBBC) and demineralized dentin matrix (DDM) [35], autogenous bone, and autogenous tooth graft [34].

For the used membranes, some studies used bioabsorbable collagen membranes [30,32,35,37], PRF [31,36], and others did not use membranes [34].

In order to evaluate and compare the reported results of the included studies. Three outcomes were compared in the included studies. Those outcomes were the percentage of the newly formed bone, the percentage of the residual graft materials, and the changes in horizontal width at the follow-up period.

### 3.3. Data Regarding the First Outcome (The Percentage of the Newly Formed Bone)

The percentage ranged from 10% of new bone in Zang et al. [36] to more than 50% of new bone formation in Castro et al. [31], Dhamija et al. [33], and Dwivedi et al. [34]. The materials that showed more than 50% of newly formed bone formation were demineralized dentin graft (ADDG) in Elfana et al. [30], leukocyte and platelet-rich fibrin (L-PRF), and advanced platelet-rich fibrin+ (A-PRF+) in Castro et al. [26], DFDBA with PRF in Dhamija et al. [33], and autogenous bone in Dwivedi et al. [34].

### 3.4. Data Regarding the Second Outcome (The Percentage of the Residual Graft Materials)

Three Studies did not report the percentage; Castro et al., 2021 [31], Dwivedi et al., 2020 [34], and Zhang et al., 2017 [36]. At the same time, those who report showed a variable range of a minimum of 1.5% for DFDBA with PRF in Dhamija et al. [33] to 13.20% for Bio-Oss Collagen, 11% for DDM in Jung et al. [35], and 13–16% for DBBM in Nart et al. [37].

### 3.5. Data Regarding the Third Outcome (The Changes in Horizontal Width at the Follow-Up Period)

The included studies reported a range from 0.6 mm in Elfana et al. [30] to 3 mm in Ridhima Dhamija et al. [33].

### 3.6. Risk of Bias

**Figure 3:**
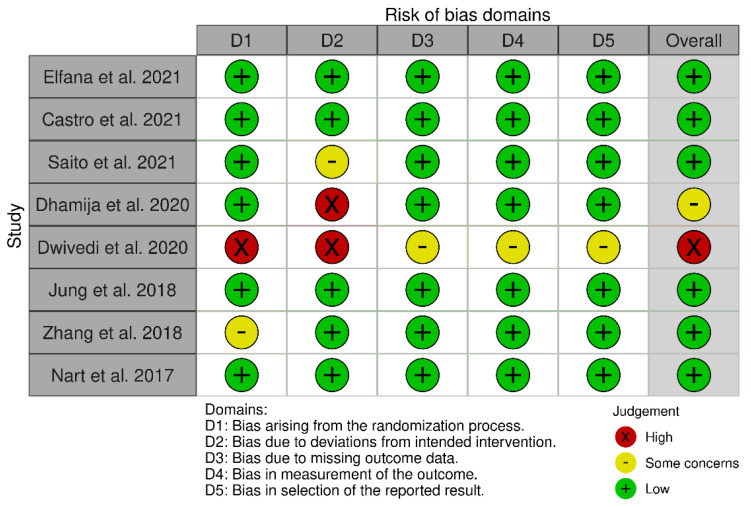
Bias analysis chart. Elfana et al. 2021 [30], Castro et al., 2021 [31], Saito et al.,2021 [32], Dhamija et al., 2020 [33], Dwivedi et al., 2020 [34], Jung et al., 2018 [35], Zhang et al., 2017 [36], and Nart et al., 2017 [37].

Risk of bias assessment showed that six studies were at “low risk”, one study showed some concerns [33], and one study was at “High risk” [34]. High risk was due to some concerns or missing information regarding D1, D2, D3, D4, and D5. Figure 3.

## 4. Discussion

The impact of different graft materials on socket preservation techniques was evaluated quantitatively and qualitatively in this systematic review.

The research question of this systematic review was “In medically fit individuals with indication for teeth extraction, what are the effects of different graft materials used in socket preservation on quantity and quality of the newly formed bone, as assessed by histology and CBCT scans”?

For clinical rationale, we included only clinical research and randomized trials using combined keywords, and no animal studies were included. The search revealed great variability in the materials used, the location of the extraction sites, and the time of evaluation. Various techniques and biomaterials have been proposed over the years to maintain the alveolar bone crest after tooth extraction [23].

In regard to the first outcome in this review, it was the percentage of the newly formed bone. Dhamija et al. [33] showed that demineralized freeze-dried bone allograft in conjunction with PRF worked as an osteoconductive material, resulting in greater and faster bone formation in more than 50% newly formed bone after 12–16 weeks compared to nongrafted sockets. 

Castro et al. [31] observed that both types of PRF (leucocyte and platelet-rich fibrin LPRF and the advanced platelet-rich fibrin+ A-PRF+) matrices seemed to promote a higher percentage of newly formed bone in comparison to non-augmented sockets, after a 3-month healing period. Regarding the percentage of socket fill, both PRF groups (L-PRF 85.2% and A-PRF 83.8%) showed a highly significant difference vs. the control (67.9%). In the control samples, a zone of unmineralized tissue was observed, while a mineralized tissue with a trabecular pattern was observed in both test groups.

On the other hand, Elfana et al. [30] histomorphometrical results showed higher quantities of newly formed bone (48.40%) in both autogenous whole tooth (AWTG) and autogenous demineralized dentin graft (ADDG) groups. Based on histological examination, both types of grafts were found to be biocompatible with the host tissues without any evidence of inflammation.

According to Dwivedi et al. [34], chair-side autogenous tooth grafts may provide better socket augmentation because they require less time, are more straightforward to prepare, have lower bone resorption speed, are osteoinductive, osteoconductive, and have osteogenetic properties, and excellent primary implant stability.

Dwivedi et al. [34] observed 34–66% of new bone formation in 40% of cases, while in 60% of cases, new bone formation ranged from 67% to 100% using the autogenous tooth graft. It was noted, however, that they encountered greater resistance during the implant placement drilling and that over 40 N cm of torque was obtained during implant insertion in 90% of patients.

The findings of Jung et al. [35] indicated that all grafted sites contained newly formed bone, remaining portions of the tooth, bone graft materials, and connective tissue. The results of this study demonstrate a significant increase in the formation of new bone in extraction sockets when recombinant human bone morphogenetic protein-2 (rhBMP-2) combined with demineralized dentin matrix (DDM) (39%) was applied into the socket at 4 months in comparison to DDM (33% alone) or DBBC (22%).

Jung et al. [35] showed that DDM has osteoinduction and osteoconduction properties and being immune-rejectable; thus, it could be an ideal material for treating hard tissue defects. 

A relatively shorter history of clinical adaptation has been seen for DDM compared to other bone substitutes or grafts. Bone and dentin have similar chemical compositions. Furthermore, approximately 90% of the dentin organic material is composed of collagen fiber, which is primarily type I collagen and performs a key role in calcification. Other organic components include growth factors, carbohydrates, lipids, and other noncollagenous proteins [45].

According to Nart et al. [37], there was no significant difference in the percentage of newly formed bone between deproteinized bovine bone minerals with (37.68%) and without collagen (33.44%). 

Based on histological analyses, Zhang et al. [36] demonstrated that new bone formation was significantly higher in the PRF group compared to the non-grafted socket group. A 9.7 ± 4.0% osteoid area was observed as opposed to a 2.8 ± 1.2% osteoid area, suggesting that PRF is capable of stimulating bone regeneration. A significant difference was observed by Saito et al. [32] in the PLGA-β-TCP group (27.0 ± 22.1%) versus the FDBA+ collagen group (38.2 ± 12.5%). The authors suggested that this is due to the slower resorption rate of β-TCP particles.

Wood et al. [46] reported mineralized tissue levels ranging from 24.63% (FDBA) to 38.42% (DFDBA) in allografts harvested approximately 20 weeks after socket preservation. While Kakar et al. [43] found an average of 21.34 ± 9.14% of newly formed bone after 20 weeks following socket preservation using β-TCP/HA for alveolar ridge preservation. 

Regarding the second outcome in this review, it was the percentage of residual graft material. In a study conducted by Dhamija et al. [33], only 1.5% of residual graft particles were found in the DFDBA +PRF group, demonstrating the significant conversion of this graft material into bone. Those findings are in agreement with those of Beck and Mealey [47] and Froum et al. [48].

According to Elfana et al. [30], there were fewer graft remnants in the ADDG group (11.45%) compared to that in the AWTG group (17.05%). A low mineral content in the ADDG group may have been responsible for these histological effects, which could have accelerated the degradation of graft particles by host cells and the subsequent release of the growth factors [49,50], which resulted in increased cell attachment and proliferation to collagen fibrils exposed [51].

As a result of the nature of the augmentation material used in Castro et al. [31], there was no residual graft material reported. A fibrin matrix in L-PRF acts as a scaffold for the entrapped cells and for growth factors that are produced by these cells, resulting in a gradual release of growth factors [52,53]. At the same time, Dwivedi et al. [34] did not report the residual graft material percentage in their study.

Jung et al. [35] observed residual graft materials as 13% for DBBC, 10% for DDM, and 11% for rhBMP-2/DDM. In this study, the demineralized dentin matrix DDM particle sizes that were used ranged from 0.5 mm to 1 mm.

The residual graft particles were 13.14% in the DBBM group and 16.00% in the DBBM-C group. Thus, Nart et al. [37] showed that both graft materials were considered suitable for ridges preservation. Radiographic and histological analysis revealed that in some cases, porcine xenograft particles did not surround newly formed bone but were encapsulated in connective tissue without any signs of inflammation, suggesting that xenograft particles may interfere with bone healing in the augmented sites.

When the particle size of the graft material is too small, resorption occurs more rapidly, while when it is too large, healing will be delayed. According to a study conducted on primates, smaller particles (100–300 m) promote osteogenesis better than large particles (1000–2000 m) [54]. 

Dhamija et al. [33] reported using DFDBA of particle size of 500–1000 µm, while Jung et al. [30] reported using DDM particles of 0.5 mm to 1 mm in diameter. Saito et al. [32] observed a higher percentage of residual graft materials in the PLGA-β-TCP group (20.5 ± 16.8%) than the FDBA+ collagen group (15.7 ± 7.0%); however, no significant difference was detected. 

Leventis et al. [55] showed that bone samples harvested after 16 weeks of socket preservation using PLGA-β-TCP demonstrated an average of 25% newly formed bone mean and 13% residual graft material, and almost 60% nonmineralized tissues. 

Considering the third outcome in this review, it was the ridge with dimensional changes. In both the control and test groups, Dhamija et al. [33] found no significant difference in ridge width decrease, with an average width loss of 3 and 2 mm at points 2 and 6 mm from the crest, respectively. Observations revealed that both groups suffered bone height and width losses, with the control sites losing more than the test sites. Moreover, both groups experienced a reduction in ridge width within 6–7 months without any significant difference between them.

As reported by Castro et al. [31], the mean change in ridge width at 1 mm from the crest was 28% for the L-PRF, A-PRF+, and non-augmented groups without any significant differences. L-PRF membranes are believed to suppress the catabolic events related to osteoclastic bone resorption, but once osteoclastogenesis has begun, they cannot reverse it [56,57].

The use of PRF matrices showed promising results in the preservation of the alveolar ridge [58,59]. Temmerman et al. [60] reported a mean change in horizontal dimension at 1 mm below the crest of 1.4 mm (23%) and 5.0 mm (51%) for the L-PRF group and control. Canellas et al. [61] showed a mean change in width at 1 mm below the crest of 0.9 mm and 2.2 mm for the L-PRF group and control group, respectively. In addition to reducing discomfort for patients, L-PRF may promote faster healing of soft tissues [62,63].

Zhang et al. [36] also found that the alveolar crest width of the non-grafted socket decreased (2.07 ± 1.7) more than the PRF group (1.05 ± 0.7). 

In contrast, Abad et al. [64] evaluated the effects of leukocyte and platelet-rich fibrin (L-PRF) on alveolar ridge preservation compared to natural socket healing. According to the CBCT results conducted after 4 months, using (L-PRF) alone did not reduce the alveolar bone loss or prevent bone regeneration for implant placement.

The comparison between AWTG and ADDG regarding the ridge width changes after 6 months revealed a small difference favoring the former, but the difference was not statistically significant. Thus, Elfana et al. [30] concluded that both types of grafts used in socket preservation had shown comparable efficacy in reducing alveolar bone changes up to 1 mm. Dwivedi et al. [34] showed an increase in ridge width by almost 1 mm after 4 months of using a mineralized tooth graft that showed a significant difference between pre and post-augmentation. 

Jung et al. [35] observed that neither the postoperative ridge width nor the level of decrease in ridge width was significantly different between the DBBC, DDM, and rhBMP-2/DDM groups at the levels of 1 and 3 mm. However, the alterations in ridge width at 5 mm showed a significant difference between DBBC (0.50 ± 0.19 mm) and DDM (0.20 ± 0.15 mm) groups. Pang et al. [65] also documented that DDM and DBBC can act as favorable biomaterials to preclude atrophic ridge alteration after tooth extraction.

Nart et al. [37] showed that sites preserved with DBBM and DBBM-C result in a similar reduction in height and width at 5 months of healing. The average ridge width changes at 1 and 3 mm from the crest for both DBBM and DBBM-C groups were (0.91, 0.35 mm) and (1.5, 0.78 mm), respectively. DBBM showed slightly fewer dimensional alterations, but this was not statistically significant.

Similar findings were observed by MacBeth et al. [66]. In which, single-rooted anterior maxillary teeth were preserved using deproteinized bovine bone (DBBM) covered with collagen membrane, socket sealed (SS), and unassisted healing. Following four months of treatment, the CBCT results showed that the DBBM group had the least change in alveolar bone width, while the SS group showed the greatest increase in alveolar ridge height. SS and DBBM groups were both able to maintain the extraction socket dimension, resulting in more clinically favorable conditions for implant placement.

After single-rooted maxillary tooth extraction, Rodrigues et al. [67] compared natural socket healing, xenograft covered with gingival-free graft, dense polytetrafluoroethylene membranes, and platelet-rich fibrin plugs. The CBCT measurements after four months showed that the xenograft group had the lowest vertical height loss, whereas both xenografts and polytetrafluoroethylene also had the lowest width loss.

Saito et al. [32] showed no statistically significant differences in the horizontal ridge width changes between the PLGA-β-TCP group and FDBA+ collagen. The average horizontal changes at 1, 3, and 5 mm were 1.3, 0.6, and 0.3 mm, respectively.

## 5. Conclusions

Regardless of the type of graft material, volumetric alteration of the post-extraction socket could not be avoided. The lack of bone substitute remnants has also been cited as an advantage of PRF in socket preservation. Demineralized dentin matrix showed favorable results when used for ridge preservation. However, the combination of rhBMP-2/DDM results in higher new bone formation compared with the use of DDM alone or deproteinized bovine bone. 

Additionally, DDM showed more bone formation and less graft remnants than the whole tooth graft. Nevertheless, chairside autogenous tooth grafting involves several steps and requires a considerable amount of time and effort. In addition, the limited quantity of autogenous grafts available is a disadvantage.

## Figures and Tables

**Table 1:** The summary of the included studies with the three main outcomes.

Study Type	Author/Year	Extraction Teeth	Study Groups	Membrane	Graft Quantity and Size	Follow-Up Period	Outcome 1 The Percentage of the Newly Formed Bone	Outcome 2 The Percentage of the Residual Graft Materials	Outcome 3 The Changes in Horizontal Width at the Follow-Up Period
RCT	Elfana et al., 2021 [30]	Singles rooted teeth	*n* = 10 Test: AWTG group Control: ADDG	Bioabsorbable collagen membrane. Open membrane approach		6 months	AWTG group 37.55 ± 8.94% ADDG group 48.40 ± 11.56%	AWTG: 18.07 ± 5.58% ADDG: 11.45 ± 4.13%	AWTG group (test) : 6.80 ± 2.61% : 0.61 ± 0.20 mm ADDG group (control) : 8.43 ± 3.66% : 0.72 ± 0.27 mm
RCT	Castro et al., 2021 [31]	Singles rooted teeth	*n* = 10 Test: L-PRF or A-PRF+ Control: unassisted socket healing.	L-PRF/A-PRF+		3 months	L-PRF: 47.7 ± 7.9% A-PRF+: 54.5 ± 5.6%	none	L-PRF group HW-1 mm: −2.2 + 1 HW-3 mm: −1.8 + −1.7 HW-5 mm: −1.2 + 0.8 A-PRF+ group HW-1 mm: −2.2 + 0.9 HW-3 mm: −1.6 + 0.9 HW-5 mm: −1.2 + 0.8
RCT	Saito et al., 2021 [32]	Multi-rooted and single Posterior teeth	*n* = 43 Group 20 Control: 23	Group1/ moldable PLGA-β-TCP Group2/ FDBA material covered with a RACD	The socket was filled with the graft material up to the bone crest	16 weeks (4 m)	Group A = 27.0 ± 22.1% Group B = 38.2 ± 12.5%	Group A = 20.5 ± 16.8% Group B = 15.7 ± 7.0%.	Group A At 3 mm: 0.61 ± 0.92 At 5 mm: 0.29 ± 0.56 Group B At 3 mm: 0.68 ± 1.59 At 5 mm: 0.56 ± 0.75
RCT	Dhamija et al., 2020 [33]	Multi rooted teeth	*n* = 15/group Test: socket preservation using DFDBA + PRF Control: no graft was placed.	DFDBA + PRF	0.5 cc vial for each test group socket DFDBA 500–1000 μ particulate	16 weeks for the histological analysis 3–6 months for CBCT	Control: 53.05 ± 15.43% Test: 57.32 ± 19.94%	1.5%	The ridge width differences before extraction and implant placement. At 2 mm and Control: 2.6 ± 0.62 Test: 3.27 ± 0.48 At 6 mm Control: 2.07 ± 0.43 Test: 3.26 ± 0.41 No significant difference was observed between the groups.
	Dwivedi et al., 2020 [34]	Single and multirooted teeth	*n* = 30 Control: autogenous bone graft Test: autogenous tooth graft	No membrane	No	3 years	34–66% of new bone formation in 40% of cases. 67–100% of bone formation in 60% of cases.	None	- Alveolar width Pre(mm.): 11.652 mm. - Alveolar Width Post (mm): 12.330
RCT	Jung et al., 2018 [35]	one or more (premolar and molar).	*n* = 10 1st Group: Bio-Oss^®^ Collagen 2nd group: DDM graft 3rd group: rhBMP-2 + DDM	DBBC and DDM	DDM ranged from 0.5 mm to 1 mm in diameter.	4 months	Ist group: 22.00 ± 11.01% 2nd group: 32.88 ± 14.48% 3rd group: 39.09 ± 15.30%	Ist group: 13.20 ± 9.79 2nd group: 10.72 ± 9.83 3rd group: 11.02 ± 12.72	Ist group: 1.14 ± 0.81 2nd group: 0.97 ± 0.39 3rd group: 0.82 ± 0.36 Alveolar bone heights and widths in the three groups (1, 2, 3) at baseline and 4 months following ridge preservation.
	Zhang et al., 2018 [36]	Multirooted teeth	*n* = 14 Control: Naturally healing sockets Tests: PRF membrane	PRF membrane	No	CBCT and histology after 3 months	Test: 9.7624 ± 4.0121% Control: 2.8056 ± 1.2094%	None	Test: 1.0500 ± 0.77862 Control: 2.0760 ± 1.67149
RCT	Nart et al., 2017 [37]	extraction of non-molar tooth	*n* = 11 Group 1: DBBM Group 2: DBBM +10% collagen (DBBM-C)	collagen membrane	No	5 months	DBBM group: 33.44 ± 17.82% DBBM-C group: 37.68 ± 13.38%	DBBM group (group 1): 13.14 ± 8.32% DBBM-C group (group 2): 16.00 ± 11.60%	DBBM group: Ridge width reduction of 9.42% at 1 mm 3.21% at 3 mm 2.53% at 5 mm DBBM-C group: Ridge width reduction of 13.83% at 1 mm 6.43% at 3 mm 4.16% at 5 mm

AWTG: autogenous whole-tooth graft, ADDG: demineralized dentin grafts, L-PRF: leukocytes platelet-rich fibrin, A-PRF: advanced platelet-rich fibrin, A-PRF+: advanced platelet-rich fibrin plus, PLGA-β-TCP: poly lactic-co-glycolic acid-coated β-tricalcium phosphate, RACD: rapidly absorbable collagen dressing, DBBM: deproteinized bovine bone mineral, FDBA: freeze-dried bone allograft, DFDBA: demineralized freeze-dried bone allograft, DBBC: deproteinized bovine bone with collagen, DDM: demineralized dentin matrix, PRF: Platelet-rich fibrin, rhBMP-2: recombinant human bone morphogenetic protein-2, DDM: demineralized dentin matrix.

## Data Availability

All data to support the findings of this study are available upon request from the corresponding author.
